# Spatiotemporal analysis of the burden of lower respiratory infections in the older adult population due to air pollution: trends from 1990 to 2021 and predictions for the next 30 years

**DOI:** 10.3389/fpubh.2025.1554694

**Published:** 2025-04-02

**Authors:** Zheng Lei, Ziguo Sun, Huiyu Li, Ji Luo, Li Zhang, Yuanjun Zhang

**Affiliations:** ^1^Emergency and Critical Care Medicine Department, Ziyang Central Hospital, Ziyang, Sichuan, China; ^2^Department of Intensive Care and Emergency Center, BaZhong Center Hospital JingKai District Branch, Bazhong, Sichuan, China

**Keywords:** lower respiratory infections, global disease burden, older adult population, air pollution, epidemiology, trend predict

## Abstract

**Background:**

Lower respiratory infections (LRI), caused by various pathogens, have significant impacts on global health. Air pollution is a major environmental factor in the development of LRI, and with ongoing urbanization and industrialization, it has become a critical public health concern. The older adult population, with declining immune function and physiological capabilities, exhibits reduced resistance to air pollution, making them a high-risk group for LRI. However, the spatiotemporal trends of LRI burden in the older adult and their association with air pollution remain understudied. This study analyzes the trends in LRI burden from 1990 to 2021 in relation to air pollution and predicts future trends from 2022 to 2050.

**Methods:**

Using data from the Global Burden of Disease (GBD 2021) database, this study examines mortality rates and disability-adjusted life years (DALY) at global, regional, and national levels from 1990 to 2021. Age-standardized rates (ASR) and estimated annual percentage changes (EAPC) were used to compare burdens across regions and time periods. A Bayesian age-period-cohort (BAPC) model was applied to predict future trends. Data analysis was conducted using R programming to explore differences in burden across genders, age groups, and socioeconomic levels.

**Results:**

From 1990 to 2021, the global burden of LRI due to air pollution generally declined, with the largest reduction in household air pollution from solid fuels. Regional differences were observed, with Asia and Africa showing increasing LRI burden from ambient particulate matter, especially in regions with lower socioeconomic development. Gender and age-specific analysis revealed that men and older populations face a higher burden, with the gap widening with age. The burden was negatively correlated with socioeconomic development. Predictions indicate a continued decrease in LRI burden due to secondhand smoke, while the LRI burden caused by ambient particulate matter and household air pollution may experience a rebound around 2035.

**Conclusion:**

While the global burden of air pollution-related LRI in older adults has decreased, regions with lower economic development, particularly in parts of Asia and Africa, continue to face high and rising burdens. Efforts should focus on strengthening the resilience of high-risk groups and implementing targeted interventions.

## 1 Introduction

Lower respiratory infections (LRI) are serious diseases caused by various pathogens such as bacteria, viruses, and fungi, primarily presenting as clinical symptoms like pneumonia and bronchitis (1). LRI is one of the leading causes of death globally, and its high pathogenicity and mortality rate make it a critical issue in the field of public health that needs urgent attention. According to the latest data from the GBD 2021 database, the global disease burden caused by LRI remains substantial. In 2021, there were 344 million cases of LRI globally, resulting in approximately 2.18 million deaths (2). The burden of LRI is influenced by various factors, including gender, age, pathogen type, individual physiological differences, and socioeconomic and environmental conditions (3–6).

Age is an important factor influencing the level of LRI burden, and the older adult population, due to the gradual decline in immune system function, coexistence of chronic diseases, and weakening of other physiological functions, becomes a high-risk group for LRI (7). Meanwhile, air pollution, as an environmental factor, has been widely confirmed as a major risk factor for various respiratory diseases. Studies have shown that long-term exposure to environmental pollutants such as particulate matter can damage the airway epithelial barrier and induce epithelial inflammation, significantly increasing the risk of lower respiratory infections (8, 9). The major air pollution exposures contributing to the LRI burden can be divided into three categories: outdoor particulate matter pollution, indoor household air pollution from solid fuels, and secondhand smoke-related air pollution. Among them, household air pollution from indoor solid fuels is more prevalent in low- and middle-income countries, where the use of solid fuels such as wood and coal releases large amounts of harmful gasses and particulate matter (10). In addition, with the global processes of urbanization and industrialization, exposure to outdoor particulate matter pollution has been on the rise in recent years (11). Numerous studies have shown that the older adult, due to their lower resistance and adaptability, are especially vulnerable to the effects of air pollution. They not only face a higher risk of LRI infection but also have a significantly higher mortality rate after infection compared to younger groups (12).

As the global population continues to age, the LRI burden in the older adult population is increasingly rising, which poses a serious threat to individual health and brings significant challenges to global health systems and societal development. Therefore, investigating the LRI burden caused by air pollution in the older adult population and its trends is of great significance for developing effective prevention strategies, optimizing public health policies, and improving the health of the older adult. This study aims to analyze the burden of LRI attributed to air pollution in the older adult population globally from 1990 to 2021, and its long-term trend changes, to gain a deeper understanding of the underlying mechanisms of air pollution-related LRI burden in the older adult population, providing scientific evidence for future public health interventions.

## 2 Method

### 2.1 Data source

All the analysis data in this study were sourced from the GBD 2021 estimated results for deaths and disability-adjusted life years (DALYs) at the global, regional, and national levels from 1990 to 2021, categorized by cause, gender, and age. Additionally, the attributable effects of 87 risk factors were specifically analyzed, and the related methods have been detailed in previous studies (13, 14). Our study focuses on the LRI disease burden attributed to air pollution in the older adult population, and we obtained data on the LRI burden due to air pollution exposure for populations aged 60 and above by gender from the GHDx online platform^1^ across all GBD geographic regions during the period from 1990 to 2021. To eliminate the impact of population structure differences across regions, we standardized the indicators by age (ASR), with the standard population structure based on the world population age distribution from GBD 2021. The formula for age standardization is as follows (Equation 1):


(1) $$ASR=\frac{\sum_{i=1}^{n}a_{i}w_{i}}{\sum_{i=1}^{n}w_{i}}$$

Where i represents the number of the current age group, n represents the total number of age groups, $a_{i}$ represents the value of the measurement indicator for the age group, and $w_{i}$ represents the number of individuals in the age group under the standard population structure.

The Socio-Demographic Index (SDI), proposed by the GBD project, is a composite indicator composed of three core indicators: years of expected education per capita, total fertility rate, and per capita income level. It can measure the level of population and social development of a country or region (15). Based on the SDI values, regions can be classified into five SDI categories: low, low-middle, middle, high-middle, and high. Through SDI comparisons, researchers and policymakers can gain a clearer understanding of the development level and health status of different regions. In GBD 2021, LRI is defined as deaths and disabilities caused by LRI, including pneumonia and bronchiolitis cases diagnosed by clinicians and self-reported cases. The GBD network uses a Bayesian-based standardization tool, integrating data from different ages, times, regions, and health sectors, to fill the gaps in countries with insufficient primary data and thus complete the global estimation of the LRI burden. All GBD estimation results are accompanied by a 95% uncertainty interval (UI), which is calculated from the 25th and 975th values of the posterior distribution. For countries with insufficient data, the UI range is wider, reflecting relatively lower estimation accuracy.

Regarding the proportion of disease attributed to risk factors, the GBD 2021 study measures this through a comparison of risk assessment methods, where the theoretical minimum risk exposure level (TMREL) and the population attributable fraction (PAF) are key measurements for risk factors (16). TMREL is a theoretical value representing the level of exposure to a risk factor at which health risks are minimized. PAF estimates the percentage reduction in disease burden if the exposure level is reduced to TMREL. That is, when the actual exposure level of a population deviates from TMREL, additional disease burden increases, and PAF quantifies the additional disease burden through this deviation. This study includes three risk factors related to air pollution exposure: ambient particulate matter pollution, household air pollution from solid fuels, and secondhand smoke. It is worth noting that TMREL has flexible values for different risk factors. Even for the same risk factor, GBD 2021 uses a time and space-variable TMREL for estimation in special circumstances, rather than a fixed global TMREL, which helps to reasonably quantify the impact levels of different risk factors (17). The specific quantification formula for the attribution of risk factors based on TMREL is as follows (Equation 2):


(2) $$PAF=\frac{\int RR\left(x\right)\cdot P\left(x\right)dx-\int RR\left(x\right)\cdot P_{TMREL}\left(x\right)dx}{\int RR\left(x\right)\cdot P\left(x\right)dx}$$

Where RR(x) represents the relative risk at a specific exposure level, P(x) represents the proportion distribution of the population exposed to the x level, and $P_{TMREL}\left(x\right)$ represents the proportion distribution of the population exposed to the TMREL level. The attributable burden (AB) is obtained by directly multiplying PAF with the disease burden (Equation 3):


(3) AB=PAF⋅B

This study primarily focuses on the burden and trend changes of Ambient particulate matter pollution, Household air pollution from solid fuels, and Secondhand smoke on the LRI burden of populations aged 60 and above. The main indicators of interest include mortality rate, DALY, Age-Standardized Mortality Rate (ASMR), Age-Standardized Disability Rate (ASDR), and their corresponding Estimated Annual Percentage Change (EAPC). It is worth noting that the GBD study used anonymized datasets, approved by the Institutional Review Board of the University of Washington, which waived the requirement for informed consent, and therefore no additional ethical approval was required.

### 2.2 Statistical analysis

The rates in GBD2021 are standardized per 100,000 population and age-standardized using the formula (Equation 1) for each age group. Additionally, linear regression methods were used to assess the trend changes of ASMR and ASDR for LRI attributable to air pollution exposure in the global, regional, and national older adult populations from 1990 to 2021, and the EAPC was estimated using the specific formulas (Equations 4, 5):


(4) y=α+βx+ε

(5) EAPC=100⋅(exp(β)−1)

In the equation, y represents ln(ASR) ; x represents the calendar year; β represents the annual average change in ASR due to air pollution. When the EAPC and the lower limit of its confidence interval (CI) are both greater than 0, the trend of ASR is considered to be increasing. When the EAPC and the upper limit of its CI are both less than 0, the EAPC is considered to show a decreasing trend. Other situations are considered to indicate a relatively stable trend.

In addition, to further explore the potential association between the burden pattern of LRI attributable to air pollution and the socio-economic and development levels, we performed a correlation analysis of the burden indicators of each region combined with SDI scores. First, based on global data and data from 21 GBD regions covering 1990 to 2021, a Pearson correlation analysis was conducted between disease burden indicators and SDI. Subsequently, based on data from 204 countries and regions, Pearson correlation analysis was used to examine the correlation between SDI scores and ASMR, ASDR, and their corresponding EAPC values.

In this study, we used the BAPC model, fitted with Integrated Nested Laplace Approximation (INLA), to predict gender-specific ASMR and ASDR in the older adult population attributable to air pollution by 2050. Studies have shown that the BAPC model has high coverage and low error rates, making it suitable for reasonable predictive analysis. The detailed methodology of the BAPC model has been elaborated in related literature (18).

All analyses were conducted in R software (version 4.3.2), with data cleaning and preparation using the tidyverse and dplyr packages; the toolkits for predicting ASMR and ASDR included BAPC, nordpred, and INLA; data visualization was performed using the ggplot2 package. All statistical tests were two-tailed, with *p*-values <0.05 considered statistically significant.

## 3 Results

### 3.1 Trends in the global and regional burden of LRIs due to air pollution among the older adult population, 1990–2021

**Figure 1:**
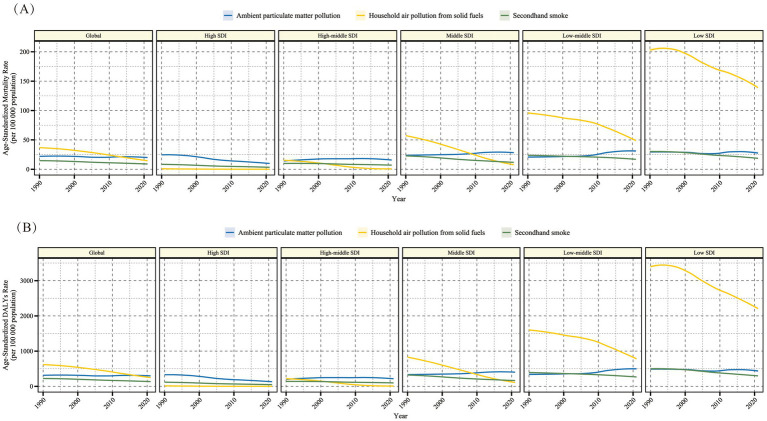
Time trends of age-standardized mortality rate (per 100,000) **(A)** and age-standardized DALY rate (per 100,000) **(B)** for older adult populations with Lower Respiratory Infections due to air pollution from 1990 to 2021, globally and across five SDI regions. DALY, Disability-Adjusted Life Years; SDI, Socio-Demographic Index. The shaded area represents the 95% uncertainty interval.

In the global context, from 1990 to 2021, the burden of LRI in older adult populations attributable to air pollution showed a declining trend. The EAPC values and 95% CI for the three risk factors were all negative (Figure 1 and Tables 1, 2). Specifically, the ASMR from Ambient Particulate Matter Pollution decreased from 22.33 (3.23, 40.17) to 18.84 (2.37, 33.89) per 100,000, and the ASDR decreased from 317.52 (45.48, 566.85) to 280.49 (35.65, 502). For Household Air Pollution from Solid Fuels, the ASMR decreased from 36.48 (5.95, 59.83) to 14.86 (2.45, 28.47), and the ASDR decreased from 613.05 (100.79, 997.72) to 254.1 (42.54, 478.96). For Secondhand Smoke, the ASMR decreased from 14.59 (4.91, 24.61) to 8.61 (2.84, 14.79), and the ASDR decreased from 222.16 (75.34, 373.06) to 130.22 (43.17, 222.5). It is worth noting that before 2010, Household Air Pollution from Solid Fuels was the major burden for older adult LRI cases due to air pollution, but after 2010, Ambient Particulate Matter Pollution became the primary burden.

**Table 1:** Age-standardized mortality rates for Lower Respiratory Infections attributable to all air pollution exposures in 1990 and 2021, and the trend of change from 1990 to 2021.

Location	Ambient particulate matter pollution			Household air pollution from solid fuels			Secondhand smoke		
	Death rate in 1990	Death rate in 2021	EAPC (1990–2021)	Death rate in 1990	Death rate in 2021	EAPC (1990–2021)	Death rate in 1990	Death rate in 2021	EAPC (1990–2021)
Global	22.33 (3.23, 40.17)	18.84 (2.37, 33.89)	−0.29 (−0.47, −0.1)	36.48 (5.95, 59.83)	14.86 (2.45, 28.47)	−2.95 (−3.19, −2.72)	14.59 (4.91, 24.61)	8.61 (2.84, 14.79)	−1.62 (−1.72, −1.52)
High SDI	24.65 (3.67, 47.09)	9.06 (1, 17.16)	−3.16 (−3.4, −2.92)	0.9 (0.09, 2.84)	0.03 (0, 0.26)	−11.29 (−11.69, −10.88)	8.35 (2.65, 14.6)	3.15 (0.98, 5.59)	−2.94 (−3.1, −2.78)
High-middle SDI	15.04 (2.36, 27.76)	15.09 (1.91, 27.17)	0.45 (0.18, 0.72)	15.4 (2.44, 28.11)	0.73 (0.02, 4.3)	−10.68 (−11.58, −9.78)	10.19 (3.38, 17.48)	6.66 (2.19, 11.65)	−1.2 (−1.33, −1.06)
Middle SDI	23.75 (3.18, 43.29)	27.03 (3.54, 49.2)	0.79 (0.64, 0.95)	56.89 (9.04, 94.66)	8.97 (0.9, 24.14)	−6.08 (−6.58, −5.58)	22.46 (7.55, 37.57)	11.52 (3.79, 19.81)	−2.21 (−2.3, −2.12)
Low-middle SDI	21.03 (2.82, 39.44)	28.83 (3.75, 54.23)	1.64 (1.3, 1.97)	94.68 (15.09, 154.63)	49.86 (7.86, 93.84)	−1.94 (−2.2, −1.68)	23.78 (7.93, 40.09)	16.37 (5.36, 28.45)	−0.99 (−1.12, −0.85)
Low SDI	30.46 (4.42, 54.19)	25.47 (3.32, 45.95)	−0.01 (−0.34, 0.31)	200.72 (34.21, 323.34)	134.35 (20.96, 220.83)	−1.3 (−1.43, −1.17)	29.83 (9.8, 51.74)	17.9 (5.71, 30.95)	−1.61 (−1.75, −1.47)
Andean Latin America	103.56 (11.84, 203.2)	66.29 (8.82, 130.36)	−1.51 (−1.77, −1.26)	84.67 (11.08, 177.03)	12.21 (0.79, 47.27)	−5.83 (−6.42, −5.23)	26.59 (8.13, 46.44)	10.64 (3.15, 20.43)	−3.13 (−3.33, −2.92)
Australasia	4.78 (0.04, 15.11)	3.36 (0.35, 6.78)	−1.01 (−1.79, −0.23)	0.07 (0, 0.77)	0 (0, 0.01)	−11.05 (−11.93, −10.16)	3.75 (1.12, 6.92)	1.15 (0.34, 2.21)	−3.3 (−3.81, −2.78)
Caribbean	25.46 (2.42, 57.43)	23.58 (2.6, 45.55)	−0.14 (−0.45, 0.18)	26.23 (4.32, 50.59)	11.34 (1.89, 22.27)	−2.39 (−2.48, −2.3)	17.07 (5.5, 29.44)	9.63 (3.11, 17.49)	−2.21 (−2.6, −1.82)
Central Asia	6.47 (0.65, 14.67)	12.95 (1.56, 23.4)	3.64 (2.94, 4.35)	7.2 (1, 15.62)	2.78 (0.3, 7.75)	−3.9 (−4.8, −3)	4.62 (1.54, 7.92)	6.11 (2.01, 10.44)	1.41 (1.12, 1.7)
Central Europe	18.96 (2.56, 35.76)	14.07 (1.7, 25.15)	−0.68 (−0.88, −0.48)	6.66 (0.62, 19.12)	0.63 (0.01, 4.44)	−8.54 (−9.16, −7.91)	8.75 (2.87, 14.9)	5.67 (1.82, 9.99)	−1.39 (−1.75, −1.04)
Central Latin America	37.52 (5.06, 72.4)	14.81 (1.79, 27.8)	−2.7 (−3.1, −2.3)	23.41 (3.49, 51)	4.81 (0.51, 13.67)	−4.87 (−5.02, −4.72)	18.21 (5.95, 30.91)	6.55 (2.09, 11.55)	−3.1 (−3.63, −2.56)
Central Sub-Saharan Africa	38.04 (4.87, 80.16)	39.86 (5.7, 77.61)	0.58 (0.33, 0.84)	279.61 (46.12, 512.6)	227.02 (37.2, 426.43)	−0.7 (−0.84, −0.56)	34.93 (10.48, 70.8)	25.37 (7.69, 50.05)	−1.13 (−1.21, −1.05)
East Asia	19.44 (2.46, 42.12)	20.44 (2.66, 37.08)	0.4 (0.12, 0.67)	73.6 (11.69, 121.29)	5.15 (0.5, 15.3)	−9.26 (−9.88, −8.63)	25.42 (8.08, 43.48)	9.53 (3.1, 17.19)	−3.59 (−3.73, −3.45)
Eastern Europe	4.35 (0.63, 8.11)	3.38 (0.4, 6.46)	−0.47 (−1.12, 0.2)	0.36 (0.03, 1.31)	0.13 (0.01, 0.61)	−5 (−6.55, −3.42)	1.55 (0.5, 2.67)	1.69 (0.52, 3.05)	0.92 (0.18, 1.67)
Eastern Sub-Saharan Africa	23.59 (3.88, 42.51)	19.95 (2.71, 36.19)	−0.11 (−0.3, 0.08)	296.39 (50.42, 476.91)	182.24 (27.8, 300.44)	−1.75 (−1.9, −1.6)	34.01 (10.89, 60.22)	16.97 (5.36, 29.98)	−2.46 (−2.63, −2.28)
High-income Asia Pacific	35.94 (2.88, 84.05)	14.14 (1.61, 27.92)	−2.57 (−2.85, −2.29)	0.38 (0.01, 2.8)	0.01 (0, 0.05)	−11.63 (−12.4, −10.86)	20.06 (6.21, 36.32)	4.53 (1.29, 8.42)	−4.47 (−4.68, −4.26)
High-income North America	17.79 (1.54, 38.31)	2.56 (0.26, 5.57)	−6.12 (−6.5, −5.74)	0.01 (0, 0.11)	0 (0, 0)	−9.53 (−9.91, −9.15)	5.33 (1.64, 9.53)	1.39 (0.42, 2.56)	−4.08 (−4.35, −3.81)
North Africa and Middle East	29.63 (4.07, 53.62)	29.88 (3.75, 52.42)	0.67 (0.49, 0.85)	18.64 (3.02, 38.02)	3.59 (0.58, 6.81)	−5.17 (−5.36, −4.99)	15.25 (5.12, 26.66)	11.04 (3.53, 19.39)	−0.53 (−0.73, −0.33)
Oceania	10.75 (0.92, 31.11)	9.56 (1.3, 24.88)	−0.22 (−0.44, 0)	108.94 (16.9, 194.91)	63.13 (8.95, 118.37)	−1.43 (−1.51, −1.34)	37.91 (12.62, 66.15)	25.83 (8.28, 46.42)	−1.06 (−1.15, −0.98)
South Asia	18.97 (2.42, 39.18)	31.81 (4.41, 58.27)	2.19 (1.75, 2.64)	93.13 (14.57, 152.93)	44.64 (6.99, 85.51)	−2.21 (−2.49, −1.92)	24.63 (8.34, 42.17)	15.93 (5.25, 28.06)	−1.3 (−1.48, −1.13)
Southeast Asia	16.6 (1.86, 36.82)	34.37 (4.25, 64.8)	3.01 (2.79, 3.23)	68.51 (11.09, 119.92)	25.63 (3.58, 56.89)	−2.6 (−3.03, −2.16)	22.27 (7.68, 39.51)	21.02 (6.45, 36.6)	0.38 (0.17, 0.6)
Southern Latin America	30.55 (3.99, 64.58)	36.26 (4.16, 71.99)	1.18 (0.77, 1.6)	18.51 (1.76, 48.27)	0.79 (0, 6.53)	−9.78 (−10.11, −9.45)	22.5 (7.31, 38.13)	16.47 (4.98, 29.51)	−0.14 (−0.44, 0.15)
Southern Sub-Saharan Africa	45.16 (5.75, 84.8)	52.38 (7.09, 96.22)	1.33 (0.82, 1.85)	68.25 (10.07, 134.37)	36.37 (5.39, 78.23)	−1.55 (−2.31, −0.79)	34.96 (11.8, 60.64)	26.07 (8.4, 45.12)	−0.42 (−0.95, 0.11)
Tropical Latin America	25.54 (1.97, 59.04)	22.1 (2.55, 43.13)	0.47 (0.13, 0.81)	22.86 (2.84, 50.81)	3.56 (0.27, 12.17)	−5.39 (−5.58, −5.19)	24.72 (8.1, 42.14)	11.09 (3.38, 20.11)	−1.85 (−2.18, −1.52)
Western Europe	24.31 (2.82, 48.92)	6.44 (0.74, 12.27)	−4.24 (−4.6, −3.87)	0.11 (0, 0.88)	0 (0, 0.03)	−10.43 (−11.05, −9.8)	6.26 (1.98, 11.02)	2.18 (0.67, 3.92)	−3.11 (−3.39, −2.83)
Western Sub-Saharan Africa	50.57 (7.59, 92.24)	48.59 (5.99, 95.83)	0.69 (0.33, 1.05)	173.82 (27.91, 290.07)	119.15 (18.83, 210.83)	−1.33 (−1.59, −1.07)	23.66 (7.7, 41.46)	14.73 (4.61, 26.03)	−1.54 (−1.75, −1.33)

**Table 2:** Age-standardized DALY rates for Lower Respiratory Infections attributable to all air pollution exposures in 1990 and 2021, and the trend of change from 1990 to 2021.

Location	Ambient particulate matter pollution			Household air pollution from solid fuels			Secondhand smoke		
	DALY rate in 1990	DALY rate in 2021	EAPC (1990–2021)	DALY rate in 1990	DALY rate in 2021	EAPC (1990–2021)	DALY rate in 1990	DALY rate in 2021	EAPC (1990–2021)
Global	317.52 (45.48, 566.85)	280.49 (35.65, 502)	−0.14 (−0.32, 0.04)	613.05 (100.79, 997.72)	254.1 (42.54, 478.96)	−2.92 (−3.15, −2.69)	222.16 (75.34, 373.06)	130.22 (43.17, 222.5)	−1.67 (−1.76, −1.57)
High SDI	329.35 (48.86, 626.4)	120.48 (13.4, 226.65)	−3.17 (−3.4, −2.94)	13.84 (1.42, 43.51)	0.46 (0, 3.92)	−11.41 (−11.81, −11)	117.04 (37.46, 203.56)	43.88 (13.83, 77.41)	−2.97 (−3.13, −2.81)
High-middle SDI	212.35 (33.2, 389.3)	207.24 (26.27, 370.11)	0.28 (0.03, 0.54)	219.39 (34.73, 400.42)	10.12 (0.23, 58.96)	−10.74 (−11.59, −9.87)	142.26 (47.36, 242.46)	91.93 (30.22, 159.56)	−1.26 (−1.39, −1.14)
Middle SDI	332.34 (44.81, 605.6)	384.63 (50.64, 696.36)	0.82 (0.67, 0.97)	821.25 (131.36, 1356.46)	127.48 (12.94, 340.85)	−6.14 (−6.61, −5.67)	316.57 (106.81, 528.94)	160.04 (52.84, 273.96)	−2.26 (−2.35, −2.18)
Low-middle SDI	344.12 (46.22, 642.57)	461.81 (60.4, 865.61)	1.5 (1.2, 1.81)	1586.23 (253.91, 2566.44)	802.21 (127.75, 1505.38)	−2.12 (−2.37, −1.86)	390.67 (130.39, 654.97)	258.13 (84.71, 447.58)	−1.17 (−1.29, −1.05)
Low SDI	505.7 (73.28, 893.88)	406.34 (53.29, 729.04)	−0.22 (−0.51, 0.07)	3373.74 (578.5, 5407.39)	2149.98 (337.51, 3532.71)	−1.52 (−1.65, −1.39)	498.15 (163.89, 864.86)	287.54 (91.57, 494.91)	−1.81 (−1.95, −1.67)
Andean Latin America	1452.1 (167.27, 2853.68)	927.07 (124.01, 1824.83)	−1.57 (−1.81, −1.33)	1196.53 (159.01, 2482.52)	175.34 (11.83, 661.71)	−5.81 (−6.38, −5.23)	376.52 (114.96, 656.88)	153.06 (44.98, 293.99)	−3.1 (−3.3, −2.9)
Australasia	59.49 (0.48, 188.12)	38.52 (4.01, 77.82)	−1.34 (−2.14, −0.53)	0.9 (0, 9.59)	0.02 (0, 0.17)	−11.38 (−12.29, −10.46)	50 (15, 92.05)	14.32 (4.22, 27.27)	−3.57 (−4.1, −3.03)
Caribbean	343.99 (33.03, 778.34)	334.21 (37.08, 648.09)	−0.03 (−0.36, 0.31)	412.19 (69.17, 791.47)	197.5 (33.32, 383.69)	−2.17 (−2.25, −2.08)	228.75 (73.86, 395.54)	134.68 (43.37, 244.29)	−2.1 (−2.49, −1.71)
Central Asia	114.25 (11.48, 257.77)	210.94 (25.32, 378.97)	3.25 (2.66, 3.85)	124.98 (17.33, 270.91)	46.25 (5.16, 127.1)	−4.14 (−5.08, −3.18)	79.72 (26.75, 136.13)	97.25 (31.93, 165.95)	1.05 (0.81, 1.3)
Central Europe	288.32 (38.83, 543.22)	216.73 (26.25, 387.23)	−0.61 (−0.79, −0.42)	100.26 (9.24, 289.93)	9.69 (0.09, 68.77)	−8.51 (−9.13, −7.88)	130.46 (42.66, 221.57)	89.05 (28.62, 156.95)	−1.19 (−1.55, −0.83)
Central Latin America	532.32 (71.72, 1028.98)	226.07 (27.2, 425.24)	−2.56 (−2.98, −2.15)	345.13 (51.59, 745.72)	73.92 (7.89, 208.3)	−4.85 (−4.99, −4.72)	256.68 (83.93, 435.48)	100.22 (32.07, 176.64)	−2.93 (−3.49, −2.36)
Central Sub-Saharan Africa	621.72 (79.14, 1318.05)	648.38 (93.21, 1263.36)	0.58 (0.33, 0.83)	4658.19 (773.42, 8551.67)	3668.05 (604.43, 6899.09)	−0.83 (−0.97, −0.68)	585.64 (176.36, 1183.8)	404.91 (121.58, 790.62)	−1.33 (−1.42, −1.25)
East Asia	251.18 (32.24, 543.46)	250.33 (32.74, 451.55)	0.23 (−0.01, 0.46)	958.72 (153.4, 1573.7)	65.4 (6.47, 192.6)	−9.3 (−9.89, −8.71)	326.31 (104.45, 557.16)	115.48 (37.46, 207.72)	−3.78 (−3.92, −3.63)
Eastern Europe	81.94 (11.89, 152.56)	64.58 (7.67, 123.17)	−0.71 (−1.49, 0.08)	6.89 (0.64, 24.77)	2.53 (0.16, 11.96)	−4.93 (−6.59, −3.24)	28.96 (9.46, 49.78)	32.65 (10.07, 59.04)	0.77 (−0.1, 1.65)
Eastern Sub-Saharan Africa	399.44 (66.26, 718.41)	318.52 (43.31, 574.99)	−0.34 (−0.52, −0.16)	4988.04 (853.69, 7982.85)	2889.05 (441.19, 4751.06)	−1.99 (−2.14, −1.84)	576.42 (183.49, 1022.66)	281.52 (89.17, 495.86)	−2.55 (−2.72, −2.39)
High-income Asia Pacific	481.15 (39.17, 1125.72)	184.96 (20.95, 363.35)	−2.63 (−2.92, −2.34)	5.17 (0.09, 38.17)	0.08 (0, 0.6)	−11.86 (−12.66, −11.06)	265.46 (82.97, 478.36)	60.29 (17.4, 111.84)	−4.43 (−4.63, −4.23)
High-income North America	246.48 (21.47, 528.31)	37.2 (3.81, 80.54)	−6 (−6.34, −5.67)	0.18 (0, 1.46)	0.01 (0, 0.07)	−9.32 (−9.7, −8.94)	80.47 (24.77, 142.67)	22.44 (6.85, 40.99)	−3.9 (−4.21, −3.59)
North Africa and Middle East	458.09 (63.27, 823.27)	448.45 (56.16, 783.24)	0.51 (0.34, 0.69)	303.38 (49.37, 607.36)	57.1 (9.24, 107.53)	−5.22 (−5.42, −5.01)	230.84 (77.35, 400.47)	161.81 (51.66, 284.02)	−0.68 (−0.85, −0.52)
Oceania	167.92 (14.39, 494.55)	148.06 (20.29, 388.17)	−0.23 (−0.45, −0.02)	1727.65 (269.52, 3094.13)	992.12 (143.07, 1865.21)	−1.46 (−1.54, −1.39)	585.69 (195.06, 1022.84)	397.96 (127.39, 718.67)	−1.08 (−1.16, −1)
South Asia	320.43 (41.38, 658.29)	508.21 (70.84, 925.89)	1.94 (1.54, 2.34)	1584.35 (249.89, 2564.05)	721.5 (115.19, 1376.02)	−2.43 (−2.69, −2.17)	409.99 (138.82, 697.78)	250.69 (82.47, 440.98)	−1.54 (−1.68, −1.4)
Southeast Asia	258.36 (29.19, 570.6)	499.86 (62.81, 941.9)	2.78 (2.57, 2.99)	1065.58 (174.61, 1845.77)	379.43 (53.01, 844.03)	−2.82 (−3.23, −2.41)	336.37 (115.99, 589.22)	302.26 (93.23, 526.78)	0.2 (0, 0.41)
Southern Latin America	433.58 (57.2, 912.3)	508.29 (58.34, 1007.26)	1.11 (0.72, 1.5)	257.73 (24.54, 674.28)	10.83 (0.02, 90.6)	−9.88 (−10.24, −9.51)	315.92 (103.18, 535.69)	237.99 (72.18, 424.53)	−0.07 (−0.35, 0.2)
Southern Sub-Saharan Africa	692.76 (87.87, 1297.99)	853.07 (115.52, 1556.56)	1.51 (0.95, 2.07)	1113.85 (169.33, 2156.99)	644.7 (97.07, 1343.61)	−1.35 (−2.08, −0.62)	540.9 (182.11, 936.67)	434.85 (140.43, 748.23)	−0.21 (−0.77, 0.36)
Tropical Latin America	381.05 (29.28, 872.6)	320.58 (37, 624.06)	0.3 (−0.04, 0.63)	320.28 (39.65, 719.67)	50.97 (3.81, 176.4)	−5.39 (−5.57, −5.2)	355.62 (117.32, 604.24)	163.78 (50.23, 297.37)	−1.83 (−2.15, −1.52)
Western Europe	305.96 (35.59, 612.66)	80.36 (9.2, 152.87)	−4.31 (−4.69, −3.94)	1.52 (0.01, 11.52)	0.04 (0, 0.38)	−10.75 (−11.37, −10.13)	82.06 (26.1, 143.99)	29.14 (9.1, 51.99)	−3.11 (−3.39, −2.83)
Western Sub-Saharan Africa	821.18 (123.45, 1493.51)	755.19 (94.89, 1494.84)	0.51 (0.16, 0.86)	2818.45 (454.7, 4687.04)	1892.03 (300.23, 3348.27)	−1.41 (−1.66, −1.15)	393.73 (127.93, 689.1)	240.07 (74.69, 423.42)	−1.64 (−1.86, −1.42)

In the five SDI regions, differences in both the composition and burden of LRI caused by air pollution were observed (Figure 1). Generally, when classified by medium SDI, higher SDI regions showed the smallest burden from Household Air Pollution from Solid Fuels, while in lower SDI regions, this risk factor represented the greatest burden. The burden from Ambient Particulate Matter Pollution and Secondhand Smoke was relatively close across all SDI regions, but a higher burden was still observed in lower SDI regions. Although the burden of LRI caused by air pollution showed a decreasing trend in almost all SDI regions, exceptions were found. For instance, Ambient Particulate Matter Pollution showed a decreasing trend only in high-SDI regions, but an increasing trend was observed in other SDI regions.

**Figure 2:**
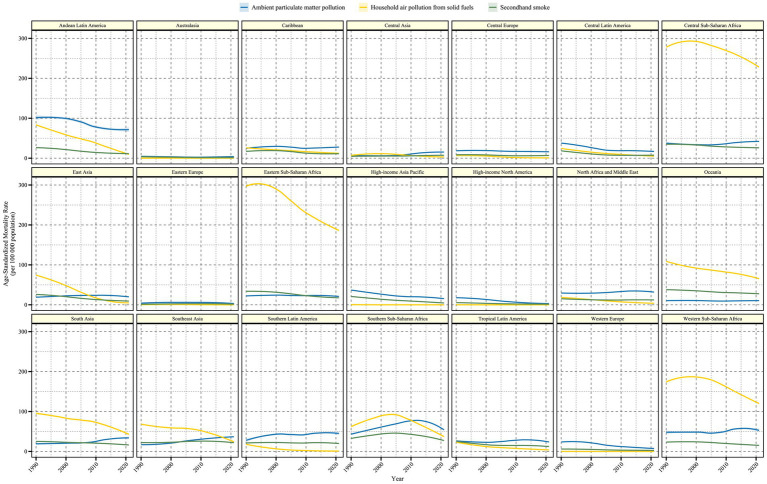
Time trends of age-standardized mortality rate (per 100,000) for older adult populations with Lower Respiratory Infections due to air pollution from 1990 to 2021 in the 21 GBD regions. The shaded area represents the 95% uncertainty interval.

**Figure 3:**
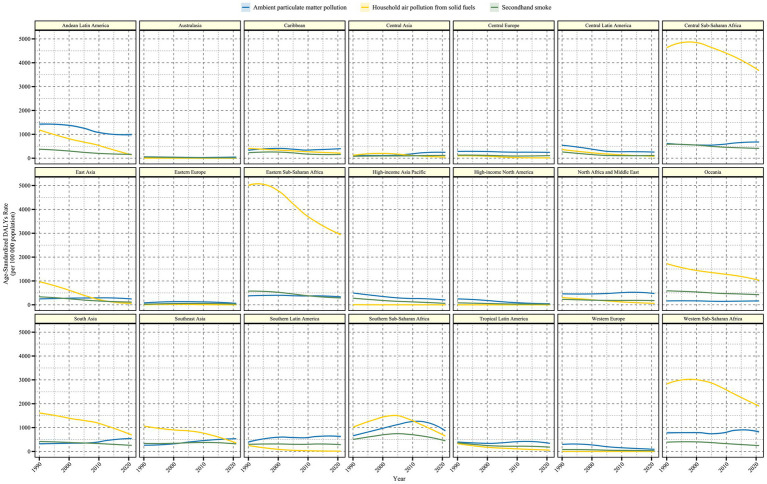
Time trends of age-standardized DALY rate (per 100,000) for older adult populations with Lower Respiratory Infections due to air pollution from 1990 to 2021 in the 21 GBD regions. DALYs, Disability-Adjusted Life Years. The shaded area represents the 95% uncertainty interval.

**Figure 4:**
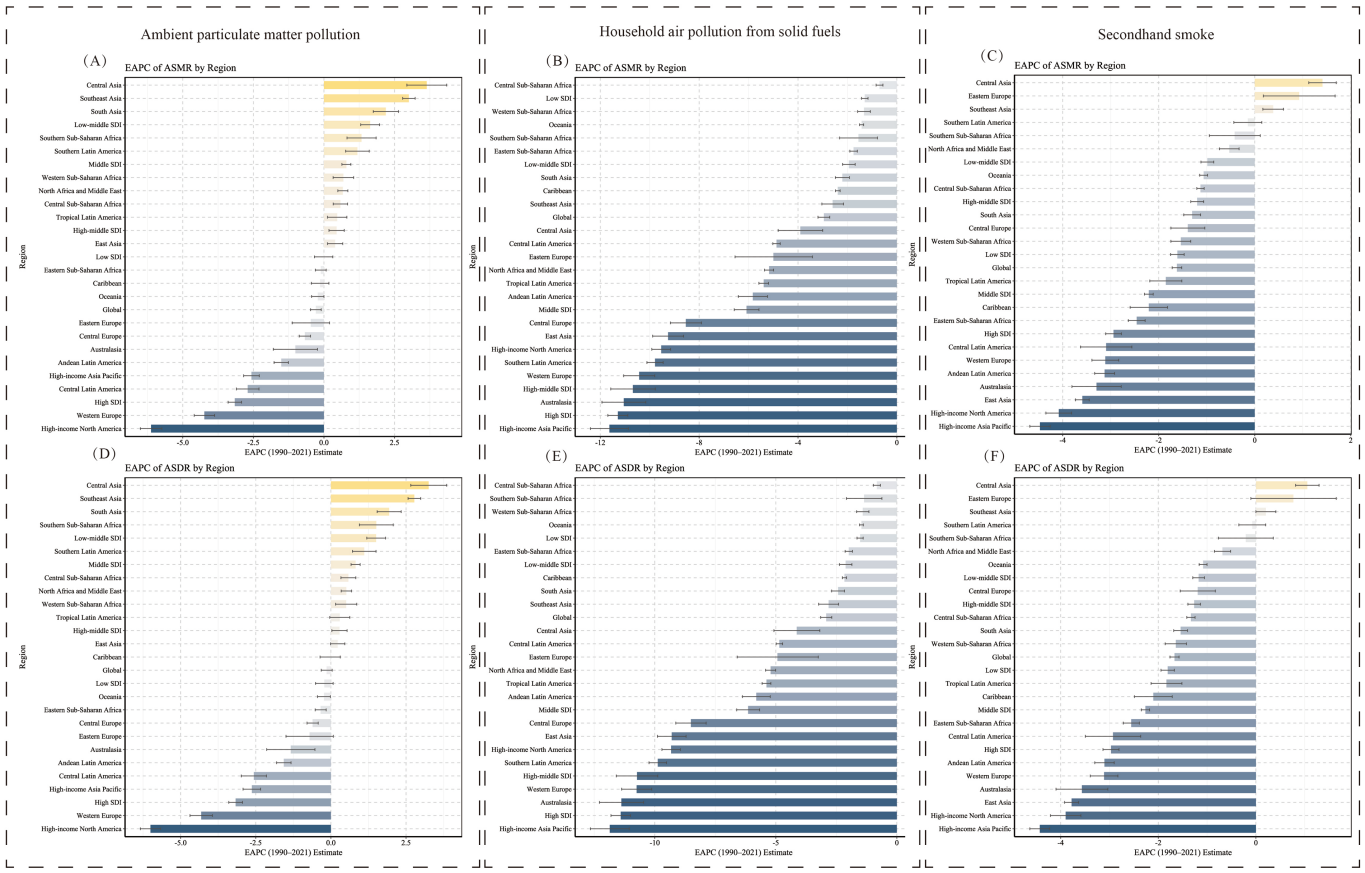
EAPC values of ASMR and ASDR (per 100,000) for older adult populations with Lower Respiratory Infections due to air pollution in the 27 GBD regions (1990–2021). **(A)** EAPC of ASMR by region for ambient particulate matter pollution. **(B)** EAPC of ASMR by region for household air pollution from solid fuels. **(C)** EAPC of ASMR by region for secondhand smoke. **(D)** EAPC of ASDR by region for ambient particulate matter pollution. **(E)** EAPC of ASDR by region for household air pollution from solid fuels. **(F)** EAPC of ASDR by region for secondhand smoke. EAPC, Estimated Annual Percentage Change; ASMR, Age-Standardized Mortality Rate; ASDR, Age-Standardized DALY Rate; DALY, Disability-Adjusted Life Years.

In the 21 regions classified by GBD, the burden and trends of LRI caused by air pollution were also examined (Figures 2, 3 and Table 1). In regions such as Central Sub-Saharan Africa, Eastern Sub-Saharan Africa, Oceania, Western Sub-Saharan Africa, and South Asia, Household Air Pollution from Solid Fuels continued to be the leading contributor to the LRI burden despite a declining trend over the past 30 years. In most regions, the burden of LRI caused by various forms of air pollution showed a decreasing trend, but regions with an increasing trend in ASMR or ASDR (EAPC >0) were identified (Figure 4). In Central Asia, South Asia, Southeast Asia, Southern Latin America, and Southern Sub-Saharan Africa, the burden of LRI caused by Ambient Particulate Matter Pollution showed an increasing trend (EAPC >1). In all regions, the burden of LRI due to Household Air Pollution from Solid Fuels showed a decreasing trend (EAPC <0). Secondhand Smoke contributed to an increasing burden of LRI only in Central Asia, Eastern Europe, and Southeast Asia (EAPC >0). Among the 21 regions, Australasia and Eastern Europe had the lowest burden of LRI caused by air pollution, with all risk factors maintaining relatively low disease burdens. However, Eastern Europe was one of the few regions where the burden from Secondhand Smoke showed an increase.

### 3.2 National burden of LRIs due to air pollution among the older adult population, 1990–2021

**Figure 5:**
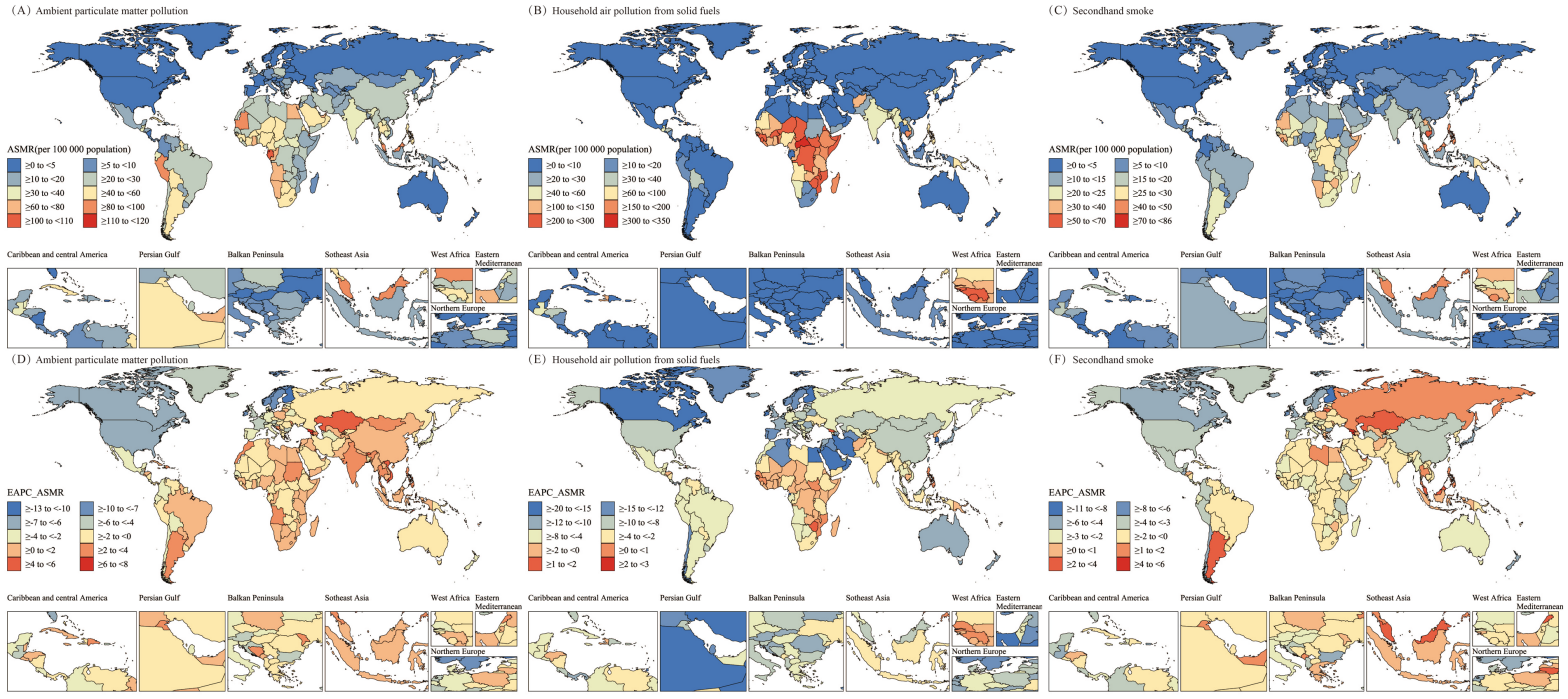
Spatial distribution of ASMR (per 100,000) and EAPC for older adult populations with Lower Respiratory Infections due to air pollution in 2021. **(A)** ASMR by region for ambient particulate matter pollution. **(B)** ASMR by region for household air pollution from solid fuels. **(C)** ASMR by region for secondhand smoke. **(D)** EAPC of ASMR by region for ambient particulate matter pollution. **(E)** EAPC of ASMR by region for household air pollution from solid fuels. **(F)** EAPC of ASMR by region for secondhand smoke. ASMR, Age-Standardized Mortality Rate; EAPC, Estimated Annual Percentage Change.

**Figure 6:**
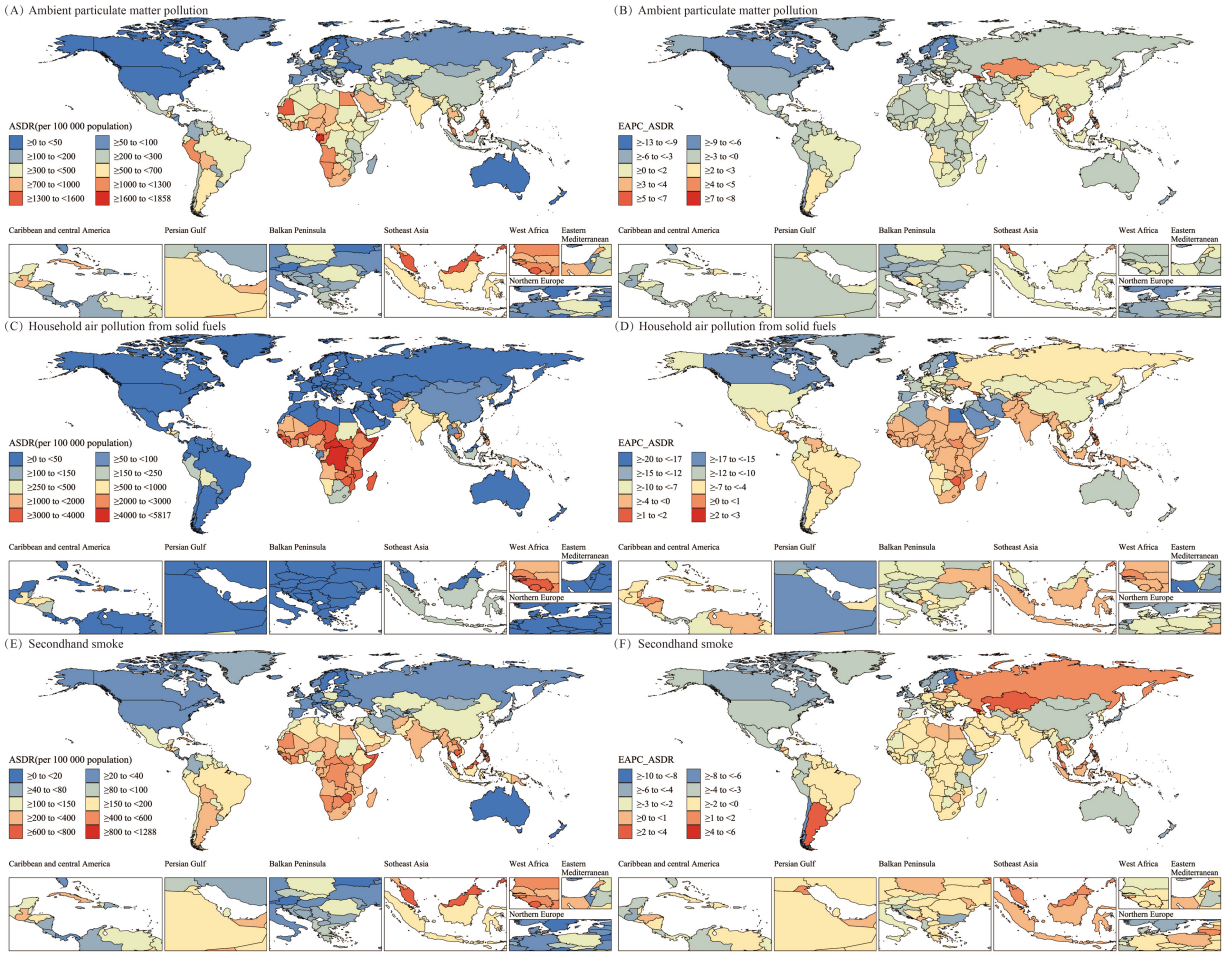
Spatial distribution of ASDR (per 100,000) and EAPC for older adult populations with Lower Respiratory Infections due to air pollution in 2021. **(A)** ASDR by region for ambient particulate matter pollution. **(B)** EAPC of ASDR by region for ambient particulate matter pollution. **(C)** ASDR by region for household air pollution from solid fuels. **(D)** EAPC of ASDR by region for household air pollution from solid fuels. **(E)** ASDR by region for secondhand smoke. **(F)** EAPC of ASDR by region for secondhand smoke. ASDR, Age-Standardized DALY Rate; DALY, Disability-Adjusted Life Years; EAPC, Estimated Annual Percentage Change.

On the level of 204 countries and regions, a world map heatmap was used to show the disease burden and trends of LRI caused by air pollution (Figures 5, 6). In 2021, for the burden of LRI caused by Ambient Particulate Matter Pollution, Equatorial Guinea, Gabon, Cabo Verde, Djibouti, and Mauritania had the highest burdens, with ASMRs over 85 and ASDRs over 1,300. For LRI attributable to Household Air Pollution from Solid Fuels, the highest burdens were observed in the Central African Republic, Somalia, Democratic Republic of the Congo, Eritrea, and Guinea-Bissau, with ASMRs greater than 265 and ASDRs exceeding 4,300. The burden of LRI due to Secondhand Smoke was highest in the Solomon Islands, Cambodia, Gambia, Nauru, and the Philippines, with ASMRs greater than 45 and ASDRs above 660. More details can be found in Supplementary Table 1.

From 1990 to 2021, the burden of LRI caused by air pollution showed a declining trend in most of the 204 countries and regions. The burden of LRI due to Household Air Pollution from Solid Fuels only increased in 8 countries and regions (with both EAPC values for ASMR and ASDR greater than 0), with the top three countries showing an upward trend being Northern Mariana Islands, Georgia, and Zimbabwe. For LRI attributable to Secondhand Smoke, the burden increased in 26 countries (with both EAPC values for ASMR and ASDR greater than 0), with the three countries with the highest increase being Georgia, Armenia, and Kazakhstan. However, in the case of LRI burden due to Ambient Particulate Matter Pollution, 80 countries or regions showed an upward trend (with both EAPC values for ASMR and ASDR greater than 0), with the top three countries or regions showing the highest increase being Georgia, Armenia, and Kazakhstan. More information regarding the EAPC values for ASMR and ASDR of the 204 countries and regions can be found in Supplementary Table 2.

### 3.3 Age-sex specific analysis of the disease burden of LRIs in the older adult caused by air pollution

**Figure 7:**
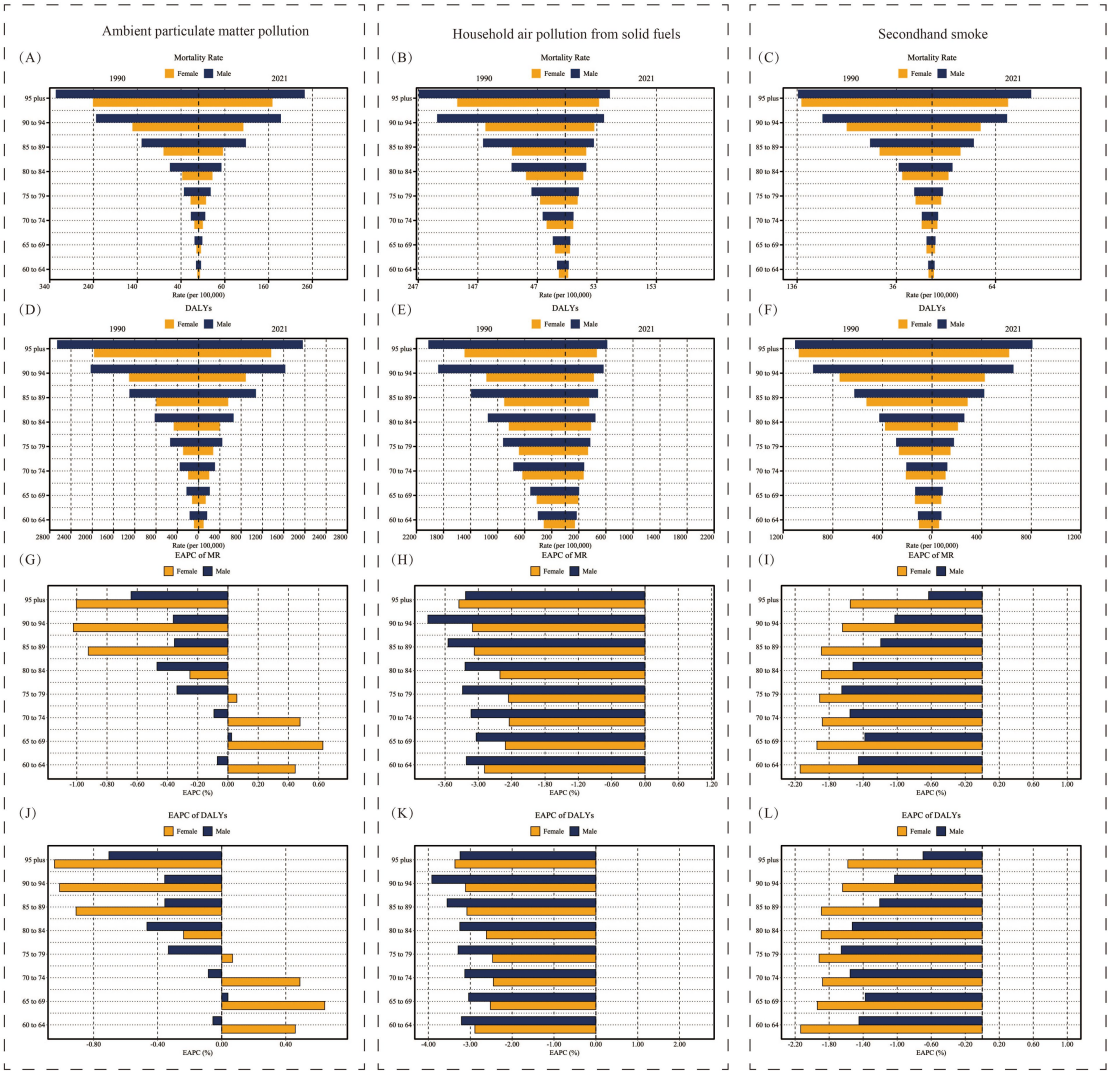
Age and sex trends of the burden of Lower Respiratory Infections due to air pollution in older adult populations in 1990 and 2021. Panels **(A–C)** show the mortality rate (per 100,000). Panels **(D–F)** show the DALY rate (per 100,000). Panels **(G–I)** show the EAPC of mortality rate. Panels **(J–L)** show the EAPC of DALY rate. Yellow bars represent females, while blue bars represent males. DALY, Disability-Adjusted Life Years; EAPC, Estimated Annual Percentage Change.

On a global scale, the burden of LRI attributed to air pollution in 1990 and 2021, including death rates, DALY rates for different age groups and genders, and their corresponding EAPC values, are shown in Figure 7. From the perspective of age groups, both death rates and DALY rates exhibit an increasing distribution, meaning that as age increases, the burden of disease also increases. The oldest age group, those aged 95 and above, bears the heaviest burden of LRI caused by air pollution. From a gender perspective, the burden of LRI caused by air pollution in older adult populations is generally higher in males than in females, and the gap between males and females widens as age increases. Compared to 1990, the disease burden in 2021 shows a downward trend in almost all age groups and gender groups. However, the burden of LRI caused by Ambient Particulate Matter Pollution in women aged 60–80 shows an upward trend, as reflected by the EAPC values (Figures 7G,J).

### 3.4 Contribution of air pollution as a risk factor to the burden of LRIs in the older adult population, 1990 and 2021

**Figure 8:**
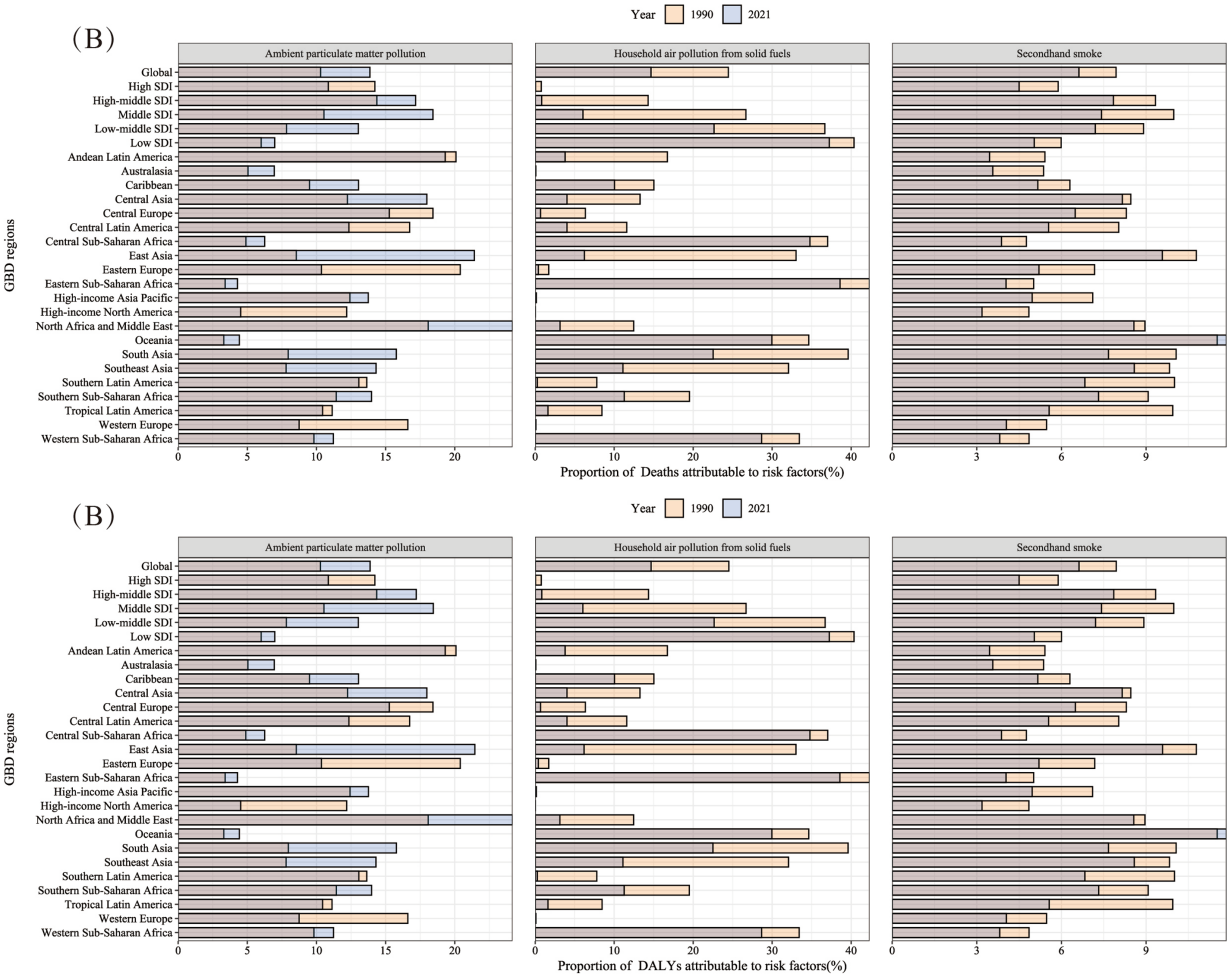
The proportion of mortality and DALY rates of Lower Respiratory Infections in 27 GBD regions in 1990 and 2021 attributable to various air pollution exposures. **(A)** Proportion of deaths attributable to ambient particulate matter pollution, household air pollution from solid fuels, and secondhand smoke. **(B)** Proportion of DALYs attributable to ambient particulate matter pollution, household air pollution from solid fuels, and secondhand smoke. DALY, Disability-Adjusted Life Years.

This analysis includes the risk factors for LRI attributed to air pollution in the GBD 2021 data. It examines the proportion of LRI attributed to various risk factors globally, within the five SDI regions, and in the 21 regions for the years 1990 and 2021 (Figure 8 and Supplementary Table 3). In 2021, the highest proportion of LRI attributed to Ambient Particulate Matter Pollution was observed in North Africa and the Middle East, and East Asia, with both regions having attribution proportions greater than 20%. The highest proportion of LRI attributed to Household Air Pollution from Solid Fuels was found in Eastern Sub-Saharan Africa and Low SDI regions, with both exceeding 35%. The highest proportion of LRI attributed to Secondhand Smoke was observed in Oceania and East Asia, with both regions exceeding 8%. Comparing the attribution proportions between 1990 and 2021 reveals that the proportion of LRI attributed to Household Air Pollution from Solid Fuels and Secondhand Smoke has decreased, except for Oceania, where the proportion of LRI attributed to Secondhand Smoke increased. However, despite a global decrease in the attribution proportion of LRI to Ambient Particulate Matter Pollution, significant differences are observed in more detailed regional divisions. Under the SDI classification, the attribution proportion increased in all regions except for High SDI. In the 21 geographical regions, the attribution proportion of Ambient Particulate Matter Pollution notably increased in many Asian and African regions. More details can be found in Supplementary Table 3.

### 3.5 The burden and trends of LRI diseases in the older adult attributed to air pollution are related to regional development levels

**Figure 9:**
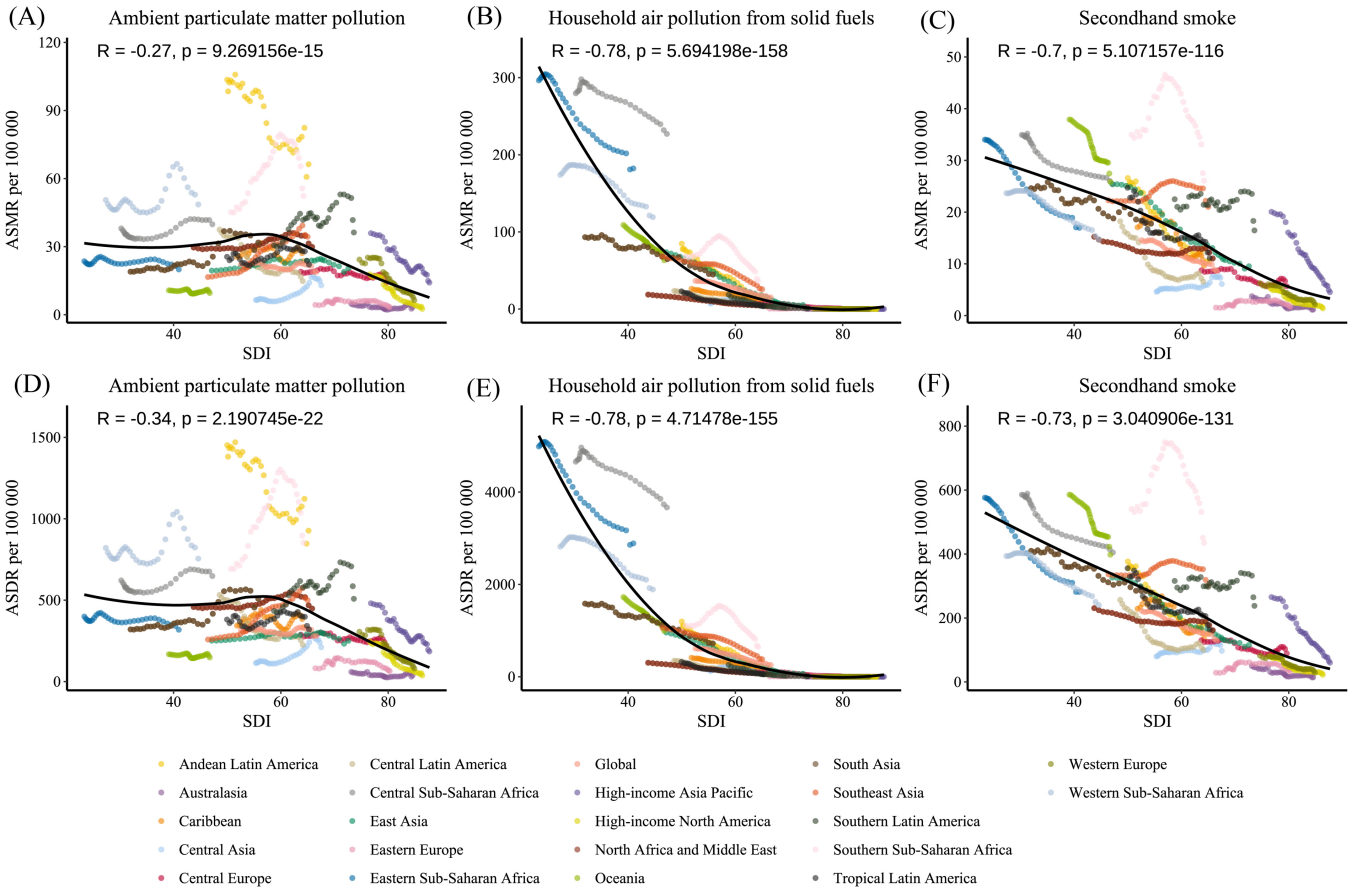
From 1990 to 2021, the age-standardized mortality rate and DALY rate of Lower Respiratory Infections caused by air pollution in 21 GBD regions, classified by the Social Demographic Index (SDI). The expected values based on SDI and burden indicators for all locations are represented by black lines. Statistical test results are shown at the top left of each subplot. **(A)** ASMR for ambient particulate matter pollution. **(B)** ASMR for household air pollution from solid fuels. **(C)** ASMR for secondhand smoke. **(D)** ASDR for ambient particulate matter pollution. **(E)** ASDR for household air pollution from solid fuels. **(F)** ASDR for secondhand smoke. ASMR, Age-Standardized Mortality Rate; ASDR, Age-Standardized DALY Rate; DALY, Disability-Adjusted Life Years.

**Figure 10:**
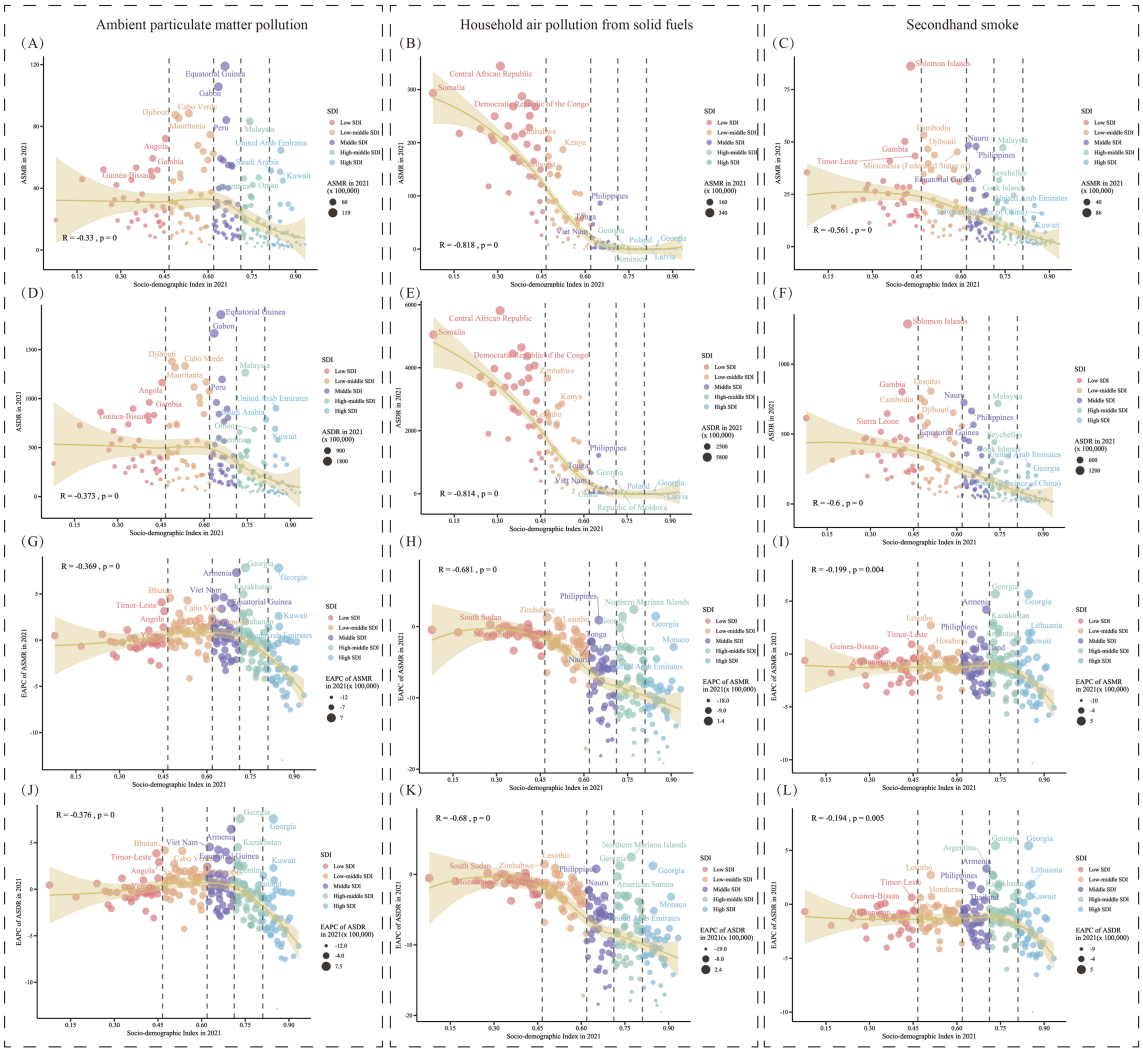
In 2021, age-standardized mortality rate and DALY rate of Lower Respiratory Infections caused by air pollution in 204 GBD countries and regions, classified by SDI. The expected values based on SDI and burden indicators for all locations are represented by black lines. Statistical test results are shown at the top left of each subplot. **(A)** ASMR for ambient particulate matter pollution. **(B)** ASMR for household air pollution from solid fuels. **(C)** ASMR for secondhand smoke. **(D)** ASDR for ambient particulate matter pollution. **(E)** ASDR for household air pollution from solid fuels. **(F)** ASDR for secondhand smoke. **(G)** EAPC of ASMR for ambient particulate matter pollution. **(H)** EAPC of ASMR for household air pollution from solid fuels. **(I)** EAPC of ASMR for secondhand smoke. **(J)** EAPC of ASDR for ambient particulate matter pollution. **(K)** EAPC of ASDR for household air pollution from solid fuels. **(L)** EAPC of ASDR for secondhand smoke. SDI, Social Demographic Index, ASMR, Age-Standardized Mortality Rate; ASDR, Age-Standardized DALY Rate; DALY, Disability-Adjusted Life Years. EAPC, Estimated Annual Percentage Change.

In the above analysis, it can be observed that the burden of LRI diseases attributed to air pollution is likely correlated with the regional development level. To further understand the correlation, the burden levels of diseases from 21 regions between 1990 and 2021 were analyzed in relation to SDI, considering both temporal and spatial aspects (Figure 9). Additionally, the correlation between current disease burden levels and SDI was examined using a finer regional classification for 204 countries and regions in 2021 (Figure 10). Ultimately, it was found that the burden of LRI diseases attributed to air pollution is strongly correlated with SDI (*p* < 0.05), with the burden decreasing as SDI increases. In the analysis of 204 countries and regions, a correlation analysis between the EAPC values of burden indicators and SDI scores was conducted, revealing a significant correlation between EAPC and SDI (Figures 10J–L). A curve was fitted for each data point, and deviations were explored to identify countries or regions where the disease burden levels were much higher than expected. The burden of LRI attributed to ambient particulate matter pollution is much higher than expected for regions with similar SDI levels, such as Andean Latin America and Southern Sub-Saharan Africa. At the same time, in Southern Sub-Saharan Africa, the LRI burden attributed to secondhand smoke is much higher than expected for the same SDI level.

### 3.6 The projected trends of gender-specific LRI burden in the older adult due to air pollution from 2022 to 2050

**Figure 11:**
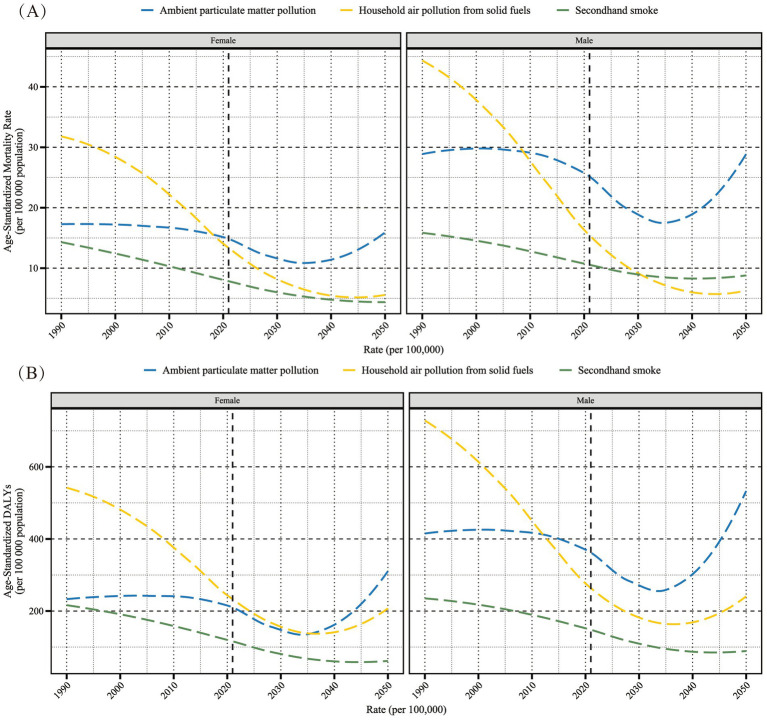
The projected trends of age-standardized mortality rate (per 100,000 people) **(A)** and age-standardized DALY rate (per 100,000 people) **(B)** for Lower Respiratory Infections attributable to air pollution in the older adult population. DALY, Disability-Adjusted Life Year.

Globally, a model was trained using data from 1990 to 2021, and BAPC was used to predict the trends of gender-specific LRI burden in the older adult caused by air pollution from 2022 to 2050 (Figure 11). It is expected that the LRI burden caused by secondhand smoke, whether measured by ASMR or ASDR, will continue to decrease and be controlled. Although the ASMR of LRI due to household air pollution from solid fuels shows a similar decreasing trend, the ASDR is predicted to rebound around 2040. The forecast for LRI burden caused by ambient particulate matter pollution shows a decrease in all genders and burden indicators in the near future, but a rebound is expected around 2035, possibly exceeding current burden levels.

## 4 Discussion

In this study, based on the GBD2021 data, we investigated for the first time the burden of LRI in the older adult caused by three major air pollution exposures and their changing trends. Overall, on both global and five major SDI scale levels, the burden of LRI in the older adult caused by air pollution has been somewhat controlled, especially household air pollution from solid fuels. Even at the scale of 204 countries and regions, only eight countries show an upward trend in the burden. This indicates that the gradual global attention to air pollution issues in recent years, along with the implementation of environmental protection policies, has achieved certain results. However, it is worth noting that household air pollution from solid fuels remains the main factor causing LRI in regions of Africa and Asia with lower socioeconomic development levels. Moreover, the burden caused by ambient particulate matter pollution is showing an increasing trend in these regions. The possible reason for this phenomenon is that these regions face difficulties in accessing energy, and their technological and economic development is lagging, leading to restrictions on the adoption of clean energy (19, 20). At the same time, due to insufficient infrastructure, many households rely on solid fuels, and this traditional heating and cooking method is influenced by social and cultural factors, making it difficult to change in the short term (21, 22). Although the burden of LRI caused by secondhand smoke has been effectively controlled and shows a decreasing trend in most regions, an upward trend is observed in Central and South Asia. It is noteworthy that although the burden of LRI caused by air pollution in Eastern Europe is at a relatively low level and well controlled, the burden caused by secondhand smoke is showing an upward trend. Therefore, we recommend taking more proactive intervention measures in these regions, especially focusing on the impact of secondhand smoke exposure on the LRI burden in the older adult.

In the study of age group and gender-specific analysis, we found that older age groups face a higher burden of LRI caused by air pollution. This result highlights the sensitivity of the older adult population to air pollution and their health vulnerability. Consistent with previous studies, older adult people have significantly lower lung function and immune defense levels than younger individuals, and they often have potential comorbidities and long-term chronic symptoms, leading to a higher LRI burden in the older adult population (23–25). In addition, changes in hormone levels are also an important factor influencing the increasing burden of LRI with age. Estrogen in women and testosterone in men both have anti-inflammatory effects. However, with aging, the sharp decline in hormone levels makes the older adult more susceptible to the effects of air pollution, triggering chronic inflammatory responses (26–28). Moreover, our study also found significant gender differences in the LRI burden caused by air pollution, with men generally bearing a higher burden than women, and this difference increases with age. Gender differences can be explained in two ways: on one hand, there is a difference in exposure to air pollution between males and females, with males likely being exposed to more pollutants; on the other hand, males are more likely to engage in other health risk factors, such as smoking and alcohol consumption, which may interact with air pollution and exacerbate the occurrence of LRI. On the other hand, physiological differences are also a key factor contributing to gender differences. Studies have shown that women’s immune systems typically have stronger immune defense functions, with more reactive T cells and B cells, allowing them to respond more effectively to pathogens in the early stages of infection (29). In contrast, men’s immune responses are slower, and their immune function tends to decline more easily compared to women, making their health more vulnerable when facing LRI induced by air pollution (30).

By analyzing the underlying patterns behind the LRI burden caused by air pollution, we found that this burden is highly correlated with the level of socioeconomic development. Specifically, we quantified the socioeconomic development of each country using the SDI value and combined it with the LRI burden indicators (mortality and DALY rates) of each country and region. By integrating the burden data of the past 30 years, we revealed a significant negative correlation between SDI values and burden levels. All statistical test results showed significance, and this finding again validated the previous hypothesis: countries with lower levels of economic development bear a higher LRI burden. The phenomenon behind this may involve the interaction of multiple factors, including energy structure, technological level, and social-cultural background. In countries with low social development levels, solid fuels are still commonly used. For example, more than 890 million people in sub-Saharan Africa still use solid fuels for cooking (31, 32). Meanwhile, outdoor particulate matter pollution sources mainly come from industrial emissions, vehicle exhaust, and thermal power generation. With the advancement of industrialization, the concentration of these pollutants shows an upward trend. In countries with higher economic development levels, the concentration of pollutants is relatively lower due to the implementation of environmental protection policies (33). By analyzing the attribution proportion of LRI caused by air pollution, we also found significant differences in the air pollution exposure patterns across different regions. In low-development regions, pollution caused by solid fuels dominates, while in more developed regions, outdoor particulate matter pollution becomes the main source of pollution. In addition, regions with low levels of social development lack effective diagnostic and treatment facilities, and the vaccination coverage for common causes of LRI is low, which may further exacerbate the LRI burden (34). In the gender-specific prediction of the LRI burden caused by three types of air pollution exposures over the next 30 years, we found that although all burdens show a declining trend in the near future, the burdens from ambient particulate matter pollution and household air pollution from solid fuels may rebound around 2035. Therefore, continuous attention to air pollution issues and the implementation of timely intervention measures are crucial.

This study is the first to combine spatial–temporal assessments of the trends of LRI burden caused by three types of air pollution exposure in the older adult population, providing scientific evidence for formulating public health intervention measures for the older adult population. However, this study also has certain limitations. First, there is difficulty in obtaining data from low-development countries, so original data is scarce, and data supplemented by mathematical modeling may have a wide uncertainty range. Secondly, the definition of LRI in the GBD2021 data may lack rigorous laboratory tests, which could lead to potential misdiagnoses and missed diagnoses (35). In addition, the definition of air pollution in GBD2021 is relatively simplified and does not cover more complex pollutant components that may exist in ambient particulate matter pollution and household air pollution from solid fuels (36). Finally, the impact of air pollution is intertwined with multiple factors, which may interact with other environmental factors (such as temperature, humidity, etc.), and this study did not further explore these interactions.

## 5 Conclusion

From 1990 to 2021, the burden of older adult LRI caused by air pollution exposure has been somewhat controlled globally, but at the regional level, regions and countries with lower levels of economic development, especially in some parts of Asia and Africa, still bear a heavier burden, and the burden is rising. Moreover, older age groups and males will bear a greater burden of air pollution-related LRI. Therefore, future efforts should prioritize strengthening the resilience of high-risk groups and taking targeted intervention measures to effectively reduce the long-term health impact of air pollution on older adult LRI.

## Data Availability

The original contributions presented in the study are included in the article/Supplementary material, further inquiries can be directed to the corresponding author.

## Footnotes

^1^ https://vizhub.healthdata.org/gbd-results/
